# Avenues of Death

**Published:** 1880-09

**Authors:** 


**AVENUES OF DEATH.**—Isaac Watts with mournful and suggestive truthfulness sang:

"Dangers stand thick through all the ground
To push us to the tomb,
And fierce diseases wait around,
To hurry mortals home.
Our life contains a thousand strings,
And dies if one be gone,
Strange that a harp of thousand strings
Should keep in tune so long."

All who died in England during 1876 were the victims of one hundred and twelve groups of disease. A hundred and twelve fatal shafts are sped about and around us, a hundred and twelve diseases are always in existence—are floating on the wings of the viewless winds—our neighbors one by one fall at our side; and at the age of forty, sixty, eighty years, we "still live," to magnify the kindness of that Eye which never slumbers or sleeps. And yet, in spite of that care, multitudes daily rush into the arms of death by inadvertence, by thoughtlessness, by inconsiderations, and by the most unwarrantable, the most reckless exposures. The same care that is expended in saving a dollar, would many a time save a holy human life. Abraham, Leviticus, Deuteronomy, or some other of the ancient notabilities sold his title, his honor, for a bowl of soup; but he was so hungry he couldn't help it. That a woman before now has walked herself into a "spell" of sickness, if not to death, in searching for a ribbon or dress-pattern of a particular shade of color, and gave as a reason, that she couldn't help it; that multitudes of them sit up sewing till near midnight, ruin their eyes, and make themselves "cross as bears" for a whole week afterwards, by the over-tax on the system, and give as an all-convincing reason, they couldn't help it: that others go into the kitchen to make pastry and cakes and pies, while Bridget stands at her ease and looks complacently on, knowing that she is paid at the rate of eight dollars a month to over-see her mistress do her own work; the said mistress getting over-heated, and exhausted, goes up-stairs, throws herself on her bed, falls asleep, gets chilled, and wakes up to be an invalid for a week or two, and gives as a reason, she couldn't help it; that at another time similar results follow from her showing a servant how to sweep a floor, make a bed, or scrub the shelves to whiteness, because she couldn't help it; that doctors live on the Avenue, and doctors' families sport faultless equipages with liveried servants and fifteen-hundred-dollar match-horses in consequence of this rather strange mode of reasoning—can not be truthfully denied, so determined do many seem to brave all providence, and to put all sense, common and uncommon, at defiance. A daughter goes to a ball or an opera, rejects gum-shoes and a shawl as precautionary. She comes home in the rain, feet wet, body chilled, with a week's illness, and reasons thus: I couldn't help it. I was there, and was obliged to come home. Ye happy husbands! how many times have you been "shut up" by this adroit mode of handling an argument on the part of your divinities? And how often patience has *not* had her perfect work when you have seen the utter falsity of the argument, but yet did not exactly "see your way clear;" in other words, hadn't sense enough to flash out the absurdity in a single, all-convincing utterance? How often this has happened, you must report yourselves. But be candid. Are you not too mad half the time to do any thing but grit the teeth, and say nothing? But these things are so, because, because we have—hem!—"hearn tell!" The real meaning of the expression, "I couldn't help it," is, I didn't choose to help it; in other words, there was an inability to act wisely, or an ignorance of cause and effect, which are equally inexcusable, if not actually criminal, often ending, as these things do, in tedious invalidism, at a ruinous expense of the husband's time and means, or in leaving whole families of motherless children to grow up uncared for, if not driven from their homes, by some designing or heartless successor; driven into stranger families; driven into ill-assorted or unwilling marriages; driven into neglect, to want, to temptation, to the acceptance of wages for accursed deeds. Health is a duty, its loss a crime.

